# Metabolism of a Selective Serotonin and Norepinephrine Reuptake Inhibitor Duloxetine in Liver Microsomes and Mice[Fn fn5]

**DOI:** 10.1124/dmd.121.000633

**Published:** 2022-02

**Authors:** Xuan Qin, John M. Hakenjos, Kevin R. MacKenzie, Mercedes Barzi, Hemantkumar Chavan, Pranavanand Nyshadham, Jin Wang, Sung Yun Jung, Joie Z. Guner, Si Chen, Lei Guo, Partha Krishnamurthy, Karl-Dimiter Bissig, Stephen Palmer, Martin M. Matzuk, Feng Li

**Affiliations:** Center for Drug Discovery, Department of Pathology & Immunology (X.Q., J.M.H., K.R.M., P.N., J.Z.G., S.P., M.M.M., F.L.), NMR and Drug Metabolism Core, Advanced Technology Cores (K.R.M., F.L.), Department of Pharmacology & Chemical Biology (K.R.M., J.W., M.M.M., F.L.), and Department of Molecular & Cellular Biology (S.Y.J., K.-D.B., F.L.), Baylor College of Medicine, Houston, Texas; Department of Pediatrics, Duke University Medical Center, Durham, North Carolina (M.B., K.-D.B.); Department of Pharmacology, Toxicology, and Therapeutics, University of Kansas Medical Center, Kansas City, Kansas (H.C., P.K.); and Division of Biochemical Toxicology, National Center for Toxicological Research/US Food and Drug Administration (FDA), Jefferson, Arkansas (S.C., L.G.)

## Abstract

Duloxetine (DLX) is a dual serotonin and norepinephrine reuptake inhibitor, widely used for the treatment of major depressive disorder. Although DLX has shown good efficacy and safety, serious adverse effects (e.g., liver injury) have been reported. The mechanisms associated with DLX-induced toxicity remain elusive. Drug metabolism plays critical roles in drug safety and efficacy. However, the metabolic profile of DLX in mice is not available, although mice serve as commonly used animal models for mechanistic studies of drug-induced adverse effects. Our study revealed 39 DLX metabolites in human/mouse liver microsomes and mice. Of note, 13 metabolites are novel, including five *N*-acetyl cysteine adducts and one reduced glutathione (GSH) adduct associated with DLX. Additionally, the species differences of certain metabolites were observed between human and mouse liver microsomes. CYP1A2 and CYP2D6 are primary enzymes responsible for the formation of DLX metabolites in liver microsomes, including DLX-GSH adducts. In summary, a total of 39 DLX metabolites were identified, and species differences were noticed in vitro. The roles of CYP450s in DLX metabolite formation were also verified using human recombinant cytochrome P450 (P450) enzymes and corresponding chemical inhibitors. Further studies are warranted to address the exact role of DLX metabolism in its adverse effects in vitro (e.g., human primary hepatocytes) and in vivo (e.g., Cyp1a2-null mice).

**SIGNIFICANCE STATEMENT:**

This current study systematically investigated Duloxetine (DLX) metabolism and bioactivation in liver microsomes and mice. This study provided a global view of DLX metabolism and bioactivation in liver microsomes and mice, which are very valuable to further elucidate the mechanistic study of DLX-related adverse effects and drug-drug interaction from metabolic aspects.

## Introduction

Duloxetine (Cymbalta, DLX) is a potent and dual inhibitor of serotonin and norepinephrine reuptake (Bymaster et al., 2001; Chalon et al., 2003; Bymaster et al., 2005). DLX is a medication mainly used for the treatment of major depressive disorder (Detke et al., 2002; Nelson et al., 2005). DLX is also approved for stress urinary incontinence (Millard et al., 2004; Guay, 2005) and peripheral neuropathic pain (Goldstein et al., 2005; Raskin et al., 2005). The safety of DLX has been well evaluated in large-scale patients across indications and healthy subjects (Wernicke et al., 2005; Gahimer et al., 2007). Although DLX has shown good efficacy, safety, and tolerance, it may cause serious adverse effects in rare cases, e.g., liver injury or hepatic failure. Clinically, DLX elevating serum alanine aminotransferase levels above 3 times the upper limit of normal occurs in ∼1% of patients. Deaths associated with DLX treatment have been described, but their relatedness to DLX needed to be verified (Hanje et al., 2006; DeSanty and Amabile, 2007; Vuppalanchi et al., 2010; Kang et al., 2011; Scanlon et al., 2016). Liver injury related to DLX usually happens within 1 to 6 months, and immunoallergic features (e.g., rash) and autoimmunity are rare (Vuppalanchi et al., 2010). Clinical studies indicated that excessive or chronic alcohol consumption escalated the risk of DLX liver toxicity. The mechanisms of DLX-induced liver toxicity remain elusive. At present, it is well appreciated that drug metabolism plays critical roles in the drug efficacy and safety. More generally, metabolism is considered as the detoxification process by modifying the chemical structure of xenobiotics, which render them readily excreted from our body. In some cases, the excess toxic intermediates (e.g., epoxides and aldehydes) formed in xenobiotic metabolism could cause various adverse effects, including toxicity (Dieckhaus et al., 2002; Baillie, 2008; Attia, 2010; Laskar and Younus, 2018). In healthy human subjects, DLX was rapidly biotransformed into a complex array of metabolites. The major pathways of DLX involve oxidation in the naphthyl ring followed by further oxidation, methylation, and conjugation of glucuronic acid and sulfate (Lantz et al., 2003). CYP1A2 and CYP2D6 are responsible for the initial oxidation step of DLX in vitro. Clinical studies revealed that CYP1A2 is the predominant enzyme contributing to DLX metabolism in vivo using potent CYP1A2 and CYP2D6 inhibitors (Skinner et al., 2003; Lobo et al., 2008), respectively. The glucuronide of 4-hydroxylated DLX and the sulfated 5-hydroxy-6-methoxylated DLX are major circulating metabolites in humans (Lantz et al., 2003). About 70% of DLX as conjugated metabolites is primarily excreted into the urine, and 20% is present in the feces as the parent drug, 4-hydroxylated-DLX, and uncharacterized metabolites. Mice are one of the frequently used animal models for the mechanistic studies of drug toxicity, as several transgenic mouse models are available (e.g., Cyp1a2-null mice). To the best of our knowledge, the metabolic fate of DLX in mice is not available, although they are very valuable for studying the mechanism of DLX-related adverse effects in mouse models. Comparison of metabolic profiles among mouse models and human subjects are indispensable for identifying the species differences.

In this study, we investigated the metabolic pathways of DLX in human liver microsomes (HLM) /mouse liver microsomes (MLM) and mice using liquid chromatography mass spectrometry (LC-MS)-based metabolomic approaches. Our previous studies demonstrated that LC-MS-based metabolomics is a rapid and effective approach to investigate drug metabolism (Li et al., 2018; Li et al., 2020), bioactivation (Li et al., 2011b; Li et al., 2014; Liu et al., 2015; Liu et al., 2016; MacKenzie et al., 2020), and toxicity (O'Connell and Watkins, 2010; Li et al., 2013; Lu et al., 2019; Zhao et al., 2019). Here, a total of 39 metabolites generated from DLX were identified, of which 13 metabolites are novel. Five *N*-acetyl cysteine (NAc) conjugated adducts associated with DLX (M34–M38) were detected and characterized in mouse urine and feces. We also identified one DLX-reduced glutathione (GSH) adduct and three NAc conjugated adducts related to DLX (M34, M35, and M38) in mouse plasma and liver. Our studies suggested that CYP2D6 and CYP1A2 are the major enzymes contributing to the formation of major DLX phase I metabolites, which are consistent with previous findings. The species differences of certain metabolites were observed in human and mouse liver microsomes. This study provides the comprehensive metabolic profiling of DLX in HLM, MLM, and mice, which may significantly contribute to the mechanistic studies of adverse effects associated with DLX metabolism and possible drug-drug interaction from metabolic aspects.

## Materials and Methods

### Materials.

DLX, [(3*S*)-N-methyl-3-(1-naphthyloxy)-3-(2-thienyl)propan-1-amine)], alpha-naphthoflavone (*α*-NF), nootkatone (NK), quercetin (QT), ticlopidine (TCP), ketoconazole, (KCZ), and sulfaphenazole (SPA) were purchased from Cayman Chemical (Ann Arbor, MI). Quinidine (Quin), formic acid, and NADPH were obtained from Sigma-Aldrich (St. Louis, MO). HLM (XTreme 200 Mixed Gender Human Liver Microsomes), MLM (Mouse Liver Microsomes), and the recombinant human CYP450s (EasyCYP Bactosomes) were purchased from XenoTech (Lenexa, KS). All solvents for liquid chromatography and mass spectrometry (LC-MS) were of the highest grade commercially available.

### Animal Treatments and Sample Preparation.

All mice (FVB mice, 2–4 months old, male) were purchased from the Jackson Laboratory and maintained under a standard 12-hour dark/light cycle with water and chow provided ad libitum. Handling was according to animal study protocols approved by the University of Kansas Medical Center Institutional Animal Care and Use Committee. Two groups of mice (*n* = 4) were orally administrated with 1 × phosphate buffered saline (PBS, 250 *µ*l for a 25 g mouse) and DLX (12 mg/kg, 1.2 mg/ml in 1 X PBS), respectively. The mice were housed separately in metabolic cages. The clinically relevant doses were administrated to mice, which were translated from the human dose (60 mg daily) (Nair and Jacob, 2016). Urine and feces were collected continually for 18 hours. Plasma and liver samples were harvested 90 minutes after the treatment of DLX (*p.o.*, 12 mg/kg) from an additional group (*n* = 3), as DLX reaches the C_max_ at 90 minutes in mice according to our pharmacokinetics study (data not published). The methods for sample preparation of urine, feces, plasma, and liver have been described in our previous report (Liu et al., 2016). Briefly, urinary samples were prepared by adding 160 *μ*l of 50% ice-cold methanol to 20 *μ*l of urine, and plasma samples were prepared by mixing 20 *μ*l of plasma with 60 *μ*l of ice-cold methanol. The sample mixtures were vortexed, centrifuged at 15,000 g for 15 minutes. Feces and liver samples were weighed and homogenized in 50% methanol (50 mg liver in 250 *µ*l; 50 mg feces in 500 *µ*l). Subsequently, 150 *µ*l of methanol was added to 50 *µ*l of the resulting mixture. The mixtures were centrifuged for 20 minutes at 15,000 g. The resulting supernatant, which was transferred to a new Eppendorf vial, was subject to a second centrifugation (15,000 g for 15 minutes). Three *µ*l of each supernatant was injected into a system combining ultra-high performance liquid chromatography (UHPLC) coupled with Q Exactive Hybrid Quadrupole-Orbitrap mass spectrometer (Q Exactive MS) for analysis.

### Metabolism of DLX in Liver Microsomes and Human Recombinant CYP450s.

Incubations were performed in 1 X PBS (pH 7.4) containing 20 *μ*M DLX and HLM or MLMs (final concentration 1.0 mg protein/ml) or 2 pmol of each cDNA-expressed P450s enzyme (control, CYP1A2, 2A6, 2B6, 2C8, 2C9, 2C19, 2D6, 2E1, and 3A4) in a final volume of 190 *μ*l. After a 5-minute preincubation at 37°C, the incubation system was fortified with 10 *μ*l of 20 mM NADPH (final concentration 1.0 mM) and incubation was continued for 40 minutes with gentle shaking. Incubations in the absence of NADPH were used as controls. Coincubations of DLX (20 *μ*M) and *α*-NF (CYP1A2 inhibitor, 6 *μ*M), or Quin (CYP2D6 inhibitor, 4 *μ*M) in HLM were performed to determine their roles in the formation of DLX major metabolites. Coincubations of DLX (20 *μ*M) with TCP (10 *μ*M, CYP2B6 inhibitor, preincubation for 20 minutes before adding DLX), or QT (30 *μ*M, CYP2C8 inhibitor), or SPA (4.0 *μ*M, CYP2C9 inhibitor), NK (10 *μ*M, CYP2C19 inhibitor), or KCZ (4.0 *μ*M, CYP3A4 inhibitor) in HLM were conducted to identify their contribution to the formation of *N*-demethyl-DLX (M4). The concentrations of CYP inhibitors used in this study were based on over 90% inhibition for their corresponding in vitro substrates that the FDA recommended. Reactions were terminated with 200 *μ*l of ice-cold methanol and vortexing for 30 seconds. After centrifugation at 15,000 g for 15 minutes, 3.0 *μ*l of each supernatant was injected onto UHPLC-Q Exactive MS system for analysis. Incubations were performed in duplicate for cDNA-expressed enzymes and in triplicate for LM experiments.

### UHPLC-Q Exactive MS Analysis.

Samples (urine, feces, plasma, and liver) from mice administered with DLX and in vitro metabolism experiments were analyzed using UHPLC coupled with Q Exactive MS (Thermo Fisher Scientific, San Jose, CA) equipped with a 100 mm × 2.1 mm BEH C-18 column (Acquity 1.7 *μ*m, Waters, Milford, MA). The column temperature was set at 40°C and the 0.3 ml/min of flow rate was used with a gradient ranging from 2% to 95% aqueous acetonitrile containing 0.1% formic acid in a 15-minute run. Q Exactive MS was operated in both positive mode and negative mode with electrospray ionization. Ultra-pure itrogen serves as the sheath (45 arbitrary unit), auxiliary (10 arbitrary unit), sweep (1.0 arbitrary unit), and collision gas. The capillary gas temperature was set at 275°C, and the capillary voltage was set at 3.7 kV. MS data in profile mode were acquired from 80 to 1200 Da. The reference ions at *m/z* 371.1012 for positive mode and 174.6592 for negative mode were used as lock masses during acquisition. The resolution was set at 140,000 and AGT target as 3e^6^. The S-lens RF level was set at 55. The MS/MS of DLX and DLX metabolites was carried out in targeted mode with an isolation width of 2 *m/z* with ramp collision energy set at 15, 20, and 35 eV.

### Data Analysis.

Xcalibur software (Thermo Fisher Scientific, San Jose, CA) was used for acquiring chromatograms and mass spectra in profile formats from *m/z* 80 to 1200. The raw file data were first input in Compound Discoverer 3.1 software (CD 3.1, Thermo Fisher Scientific, San Jose, CA), followed by processing with untargeted metabolic workflow. The mass tolerance for alignment was set at 5 ppm and retention time shift at 1.0 minute. Minimal intensity was set at 300,000 for compound detection. Multivariate data matrix was extracted from CD 3.1. in Excel format included retention times, exact masses, and peak areas of each ion in each analyzed sample. (User guide: https://assets.thermofisher.com/TFS-Assets/CMD/manuals/man-xcali-98120-compound-discoverer-user- manxcali98120-en.pdf). Extracted data matrices were then exported into SIMCA14 (Umetrics, Kinnelon, NJ) for multivariate data analysis (Cazanave et al., 2009). Orthogonal projection to latent structures-discriminant analysis (OPLS-DA) was performed on Pareto-scaled data (Worley and Powers, 2013). For chemometric analysis, the matrix data from *m/z* 100 to 750 were processed. Statistical analysis was performed using student’s independent *t* test. All the presented data are as mean ±S.E.M.

## Results

### Profiling DLX Metabolism in Mice Using a Metabolomic Approach.

**Fig. 1.**
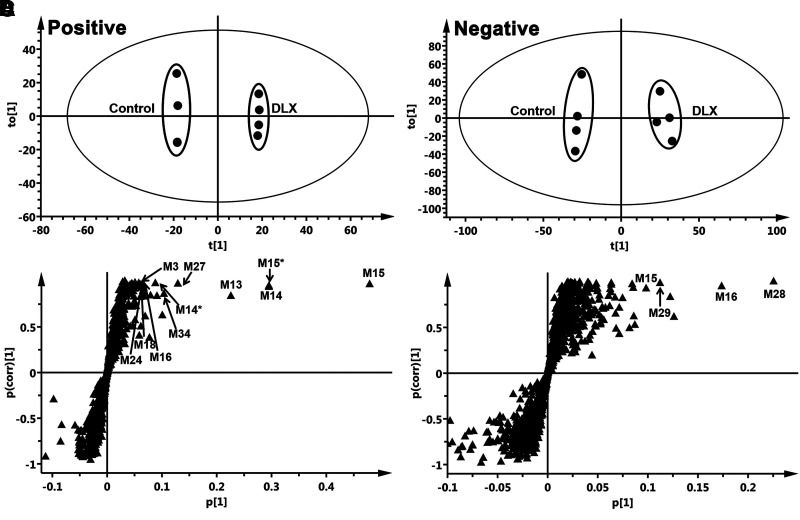
Metabolomic analysis of ions from the control and DLX-treated mouse urine samples. Wild-type mice (*n* = 4) were treated with 12 mg/kg DLX (*p.o.*). Urine and feces were collected continually for 18 hours after treatment. The samples were analyzed using UHPLC-Q Exactive MS in both positive and negative modes. (A, B) Separation of control and DLX-treated mouse urinary metabolomes generated from positive and negative modes in OPLS-DA score plots, respectively. The t[1] and to[1] values represent the score of each sample in principal component 1 and 2, respectively. (C, D) Loading S-plot generated by OPLS-DA analysis of mouse urinary metabolomes generated from positive and negative modes. The X-axis is a measure of the relative abundance of ions, and the Y-axis is a measure of the correlation of each ion to the model. These loading plots represent the relationship between variables (ions) in relation to the first and second components present in the OPLS-DA score plot. DLX and its metabolites were labeled in S-plots. The number of ions (metabolite identification) was accordant with that in Table 1. *, in-source fragment.

**Fig. 2.**
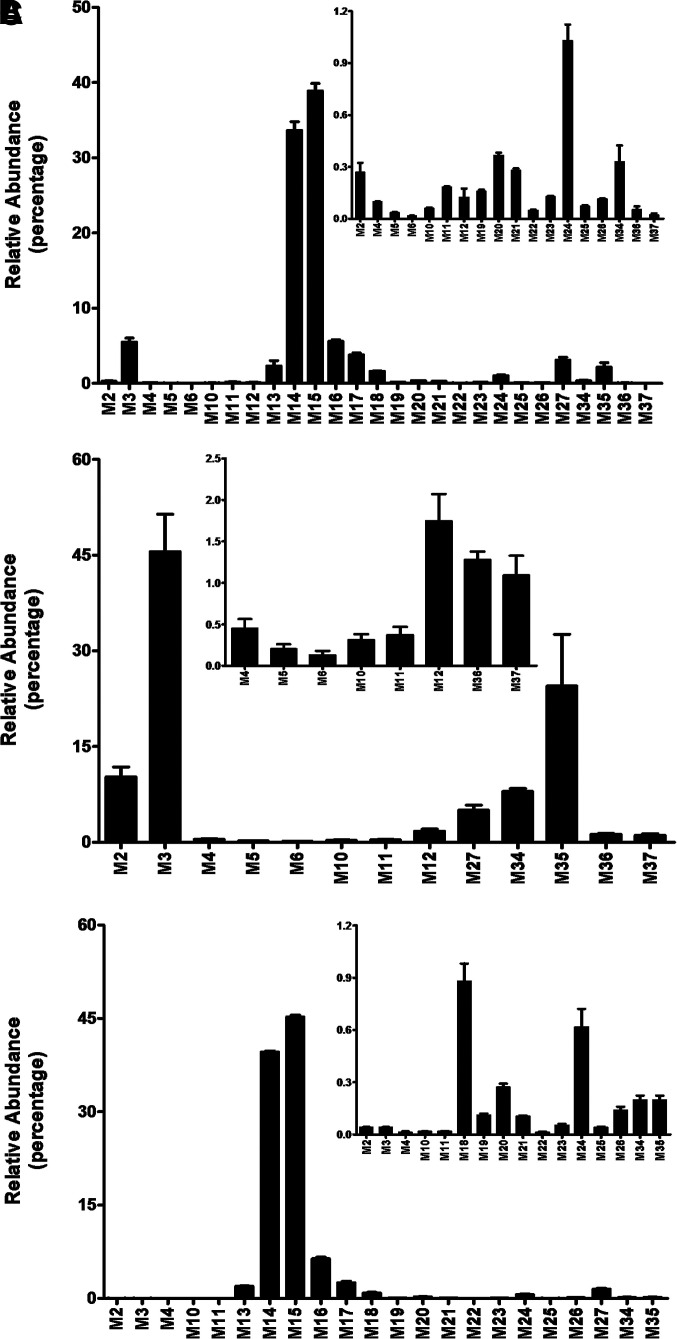
Relative abundance of metabolites of DLX in mouse urine, feces, and plasma (positive mode). Urine and feces from mice were collected continuously over the 18 hours after treatment of analysis. Plasma and liver were collected 90 minutes after the treatment. The samples were analyzed using UHPLC-Q Exactive MS in positive mode. The relative quantification was conducted based on the peak area. The overall abundance of metabolites was set as 100% in each sample. (A) Relative abundance of metabolites in urine. (B) Relative abundance of metabolites in feces. (C) Relative abundance of metabolites in plasma. All the data are expressed as mean ±SEM (urine and feces, *n* = 4; plasma, *n* = 3).

**Fig. 3.**
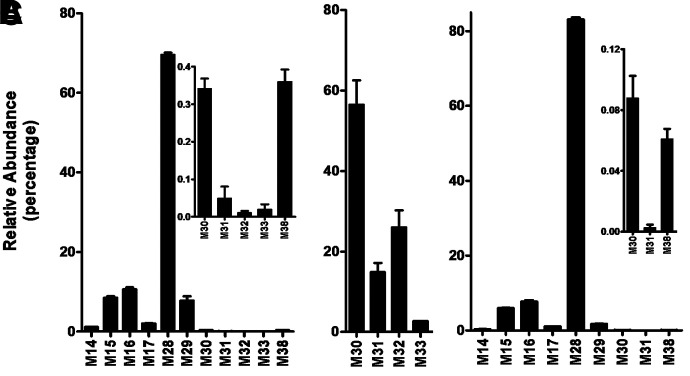
Relative abundance of metabolites of DLX in mouse urine, feces, and plasma (negative mode). The samples in Fig. 2 were analyzed using UHPLC-Q Exactive MS in negative mode. The relative quantification was conducted based on the peak area. The overall abundance of metabolites was set as 100% in each sample. The data are expressed as mean (urine and feces, *n* = 4; plasma, *n* = 3). (A) Relative abundance of metabolites in urine. (B) Relative abundance of metabolites in feces. (C) Relative abundance of metabolites in plasma. All the data are expressed as mean ±SEM.

The results of the chemometric analysis on the ions produced from the UHPLC-Q Exactive MS analysis of urine samples from control and DLX-treated mice are shown in Fig. 1. The mouse urinary metabolomes from both positive and negative modes were analyzed. The principal component analysis revealed two clusters corresponding to the control and DLX-treated groups (Fig. 1A, positive; Fig. 1C, negative). OPLS-DA generated the S-plot (Fig. 1B, positive; Fig. 1D, negative), which displays the ion contribution to the group separation. The top-ranking ions contributing to group separation are DLX and its metabolites and marked in the corresponding S-plots. DLX and its metabolites were excreted in both urine and feces, but largely in the urine (M2–M6 and M10–M38) (Figs. 2A, 3A, and Table 1). The metabolites M2–M6, M10–M12, M27, and M30–M37 were found in feces (Figs. 2B, 3B, and Table 1). Thirteen DLX metabolites were formed from phase I reactions in LM, and nine of them were also detected in mice (Table 1). Additionally, 26 phase II metabolites were identified in mice, including 15 DLX-glucuronides (M13–M26, M29), 5 O+DLX-sulfates (M28, M30–M33), 4 DLX-NAc adducts (M34–M37), one naphthol-NAc-sulfate (M38), and one DLX-GSH adduct (M39). Totally, we identified 39 DLX metabolites in liver microsomes and mice, and 13 of them are novel.

**TABLE 1:** Summary of DLX metabolites in liver microsomes and mice

RT (min)	Observed *m/z* [M+H]^+^	Calculated *m/z* [M+H]^+^	Mass Error (ppm)	Predicted Molecular Formula	Identification	Metabolite ID	Source
8.05	298.1259	298.1260	−0.33	C_18_H_19_NOS	Duloxetine	DLX	HLM, MLM, U, F, S, L
6.42	314.1208	314.1209	−0.32	C_18_H_19_NO_2_S	O+DLX	M1	HLM, MLM
6.56	314.1208	314.1209	−0.32	C_18_H_19_NO_2_S	O+DLX	M2	HLM, MLM, U, F, S, L
6.75	314.1209	314.1209	0.00	C_18_H_19_NO_2_S	O+ DLX	M3	HLM, MLM, U, F, S, L
7.87	284.1104	284.1104	0.00	C_17_H_17_NOS	DLX-CH_3_	M4	HLM, MLM, U, F, S, L
6.57	300.1052	300.1053	−0.32	C_17_H_17_NO_2_S	O+DLX-CH_3_	M5	HLM, MLM, U, F, L
6.41	300.1054	300.1053	0.32	C_17_H_17_NO_2_S	O+DLX-CH_3_	M6	HLM, MLM, U, F, L
5.47	330.1157	330.1158	−0.30	C_18_H_19_NO_3_S	2O+DLX	M7	HLM
6.11	330.1158	330.1158	0.00	C_18_H_19_NO_3_S	2O+DLX	M8	HLM
6.58	330.1159	330.1158	0.30	C_18_H_19_NO_3_S	2O+DLX	M9	HLM, MLM
4.69	332.1314	332.1315	−0.30	C_18_H_21_NO_3_S	2O+DLX+2H	M10	HLM, MLM, U, F, S, L
4.88	332.1314	332.1315	−0.30	C_18_H_21_NO_3_S	2O+DLX+2H	M11	HLM, MLM, U, F, S, L
6.61	328.1000	328.1002	−0.60	C_18_H_17_NO_3_S	2O+DLX-2H	**M12**	HLM, MLM, U, F, L
5.17	474.1583	474.1581	0.42	C_24_H_27_NO_7_S	DLX+Glu	**M13**	U, S, L
4.81	490.1531	490.1530	0.20	C_24_H_27_NO_8_S	O+DLX+Glu	M14	U, S, L
5.12	490.1532	490.1530	0.41	C_24_H_27_NO_8_S	O+DLX+Glu	M15	U, S, L
5.19	490.1530	490.1530	−0.00	C_24_H_27_NO_8_S	O+DLX+Glu	M16	U, S, L
5.88	490.1531	490.1530	0.20	C_24_H_27_NO_8_S	O+DLX+Glu	M17	U, S, L
4.93	476.1375	476.1374	0.21	C_23_H_24_NO_8_S	O+DLX-CH_3_+Glu	M18	U, S, L
4.96	506.1482	506.1479	0.59	C_24_H_27_NO_9_S	2O+DLX+Glu	M19	U, S, L
4.51	506.1481	506.1479	0.40	C_24_H_27_NO_9_S	2O+DLX+Glu	M20	U, S, L
4.31	508.1640	508.1636	0.79	C_24_H_29_NO_9_S	2O+DLX+2H+Glu	M21	U, S, L
4.18	508.1639	508.1636	0.59	C_24_H_29_NO_9_S	2O+DLX+2H+Glu	M22	U, S, L
4.08	508.1637	508.1636	0.20	C_24_H_29_NO_9_S	2O+DLX+2H+Glu	M23	U, S
4.69	520.1638	520.1636	0.38	C_25_H_29_NO_9_S	2O+DLX+CH_3_+Glu	M24	U, S, L
5.13	520.1637	520.1636	0.19	C_25_H_29_NO_9_S	2O+DLX+CH_3_+Glu	M25	U, S
5.59	520.1638	520.1636	0.38	C_25_H_29_NO_9_S	2O+DLX+CH_3_+Glu	**M26**	U, S, L
2.52	172.0790	172.0791	−0.58	C_8_H_13_NOS	Alcohol	M27	HLM, MLM, U, F, S, L
6.51	223.0068	223.0071	−1.35	C_10_H_8_O_4_S	Naphthol+SO_3_H	M28*	U, S, L
6.48	319.0821	319.0823	−0.63	C_16_H_16_O_7_	Naphthol+ Glu	M29*	U, S, L
6.04	392.0629	392.0632	−0.77	C_18_H_19_NO_5_S_2_	O+DLX+SO_3_H	M30*	U, F, S, L
6.28	392.0628	392.0632	−1.00	C_18_H_19_NO_5_S_2_	O+DLX+SO_3_H	M31*	U, F, S, L
6.43	392.0628	392.0632	−1.00	C_18_H_19_NO_5_S_2_	O+DLX+SO_3_H	M32*	U, F, L
7.31	392.0631	392.0632	−0.26	C_18_H_19_NO_5_S_2_	O+DLX+SO_3_H	M33*	U, F
4.82	477.1513	477.1512	0.21	C_23_H_28_N_2_O_5_S_2_	O+DLX+2H+NAc	**M34**	U, F, S
4.91	477.1512	477.1512	0.00	C_23_H_28_N_2_O_5_S_2_	O+DLX+2H+NAc	**M35**	U, F, S, L
5.53	477.1514	477.1512	0.42	C_23_H_28_N_2_O_5_S_2_	O+DLX+2H+NAc	**M36**	U, F
6.48	459.1407	459.1407	0.00	C_23_H_26_N_2_O_4_S_2_	DLX+NAc	**M37**	U, F
3.58	402.0316	402.0323	−1.74	C_15_H_17_NO_8_S_2_	O+Naphthol+ 2H+NAc+SO_3_H	M38*	U, S, L
3.99	621.2053	621.2047	0.97	C_28_H_36_N_4_O_8_S_2_	O+DLX+2H+GSH	M39	L

DLX, duloxetine; F, feces; Glu, glucuronic acid; GSH, glutathione; HLM, human liver microsome; L, liver; MLM, mouse liver microsome; NAc, N-acetyl cysteine; O+, monohydroxylation; 2O+, dihydroxylation; 2O+2H, monohydroxylation + hydrogenation; OSO_3_H, sulfate; RT, retention time; S, serum; U, urine.

*Metabolites detected in negative ionization mode [M-H]^-^.

Cyan indicated the novel metabolites.

### Excretion of DLX and Its Metabolites in Mice and Biotransformation of DLX in Liver Microsomes.

In mouse urine, a total of 34 metabolites were identified in the analysis of data from both positive and negative modes. The analysis of urinary data from positive mode revealed 27 metabolites, and four O+DLX+glucuronides (M14–M17, 82%) are the predominant metabolites. O+DLX (M3, 5.5%), DLX+glucuronide (M13, 2.3%), O+demethylated-DLX+glucuronide (M18, 1.6%), alcohol (M27, 3.0%), and DLX-NAc adduct (M35, 2.1%) are secondary to O+DLX+glucuronides (Fig. 2A). The analysis of urinary data from negative mode uncovered 11 metabolites including four O+DLX+glucuronides (M14–M17, 22%) that were also detected in positive modes, 1-naphthol+sulfate (M28, 69.5%), 1-naphthol+glucuronide (M29, 7.8%), four O+DLX+sulfates (M30–M33, 0.4%) and 1-naphthol-NAc-sulfate (M38) (Fig. 3A). In feces, 13 metabolites were observed in the analysis of data from positive mode including two O+DLX (M2–M3, 55.8%), demethylated-DLX (M4, 0.45%), two O+demethylated-DLX (M5–M6, 0.33%), three dihydroxylated-dehydrorogenated-DLX (M10–M12, 2.43%), alcohol (M27, 5.0%), four DLX-NAc adducts (M34–M37, 34.8%), which are presented in Fig. 2B. Only four O+DLX+sulfates (M30–M33) were detected in negative mode (Fig. 3B). The relative abundances of DLX metabolites in mouse urine and feces were shown in Figs. 2A and 2B (positive mode) and Figs. 3A and 3B (negative mode). Additionally, 22 circulating metabolites were observed in the mouse plasma (positive mode), and their relative abundance was displayed in Fig. 2C. Among these metabolites, O+DLX+glucuronides (M14–M17, 93.75%) are the predominant circulating metabolites (Fig. 2C) in positive mode. The analysis of plasma data from negative mode revealed nine metabolites (Fig. 3C): one leading metabolite 1-naphthol+sulfate (M28, 83%), four O+DLX+sulfates (M30–M33, 15%), one 1-naphthol+glucuronide (M29, 1.7%), two trace amounts of O+DLX+sulfates (M30 and M31, 0.09%), and 1-naphthol-NAc-sulfate (M38). In mouse liver, monohydroxylated metabolites (O+DLX, M2, and M3, 56.3%) are the major metabolites, followed by three O+DLX+glucuronides (M14–M16, 21.9%), demethylated-DLX (M4, 9.6%), alcohol (M27, 5.3%), and DLX-NAc adducts (M34 and M35, 2.58%) (Supplementary Fig. 1A). 1-Naphthol+sulfate (M28, 68.6%) is the predominant metabolite in liver sample analysis in negative mode. Four O+DLX+glucuronides (M14–M17, 28.9%), one 1-naphthol+glucuronide (M29, 0.9%), and three O+DLX+sulfates (M30–M32, 1.52%) were presented in the liver samples (Table 1 and Supplementary Fig. 1B). In the liver, one DLX-GSH adduct (M39) was detected together with one DLX-NAc adduct (M35).

**Fig. 4.**
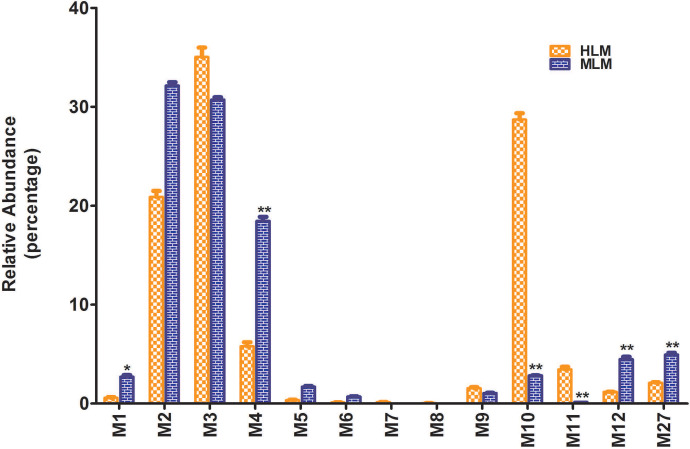
Relative abundance of metabolites of DLX in human and mouse liver microsomes. Incubations were conducted in 1X phosphate-buffered saline (1 X PBS, pH 7.4), containing 20 *µ*M DLX, 0.2 mg HLM in a final volume of 190 *µ*l. After 5 minutes of preincubation at 37°C, the reaction was initiated by adding 10 *µ*l of 20 mM NADPH (final concentration 1.0 mM) and continued for 40 minutes with gentle shaking. The relative quantification was conducted based on the peak area. The overall abundance of metabolites was set as 100% in each sample. The data are expressed as mean ±SEM (*n* = 3). Statistical analysis between the groups was conducted using student’s independent *t* test. *P < 0.05, **P < 0.01.

Incubations of HLM and MLM with DLX revealed a total of 13 stable metabolites (Table 1) and four of them were discovered for the first time. M2 (HLM, 21%; MLM, 32%) and M3 (HLM, 35%; MLM, 31%) are the primary metabolites in both HLM and MLM (Fig. 4). In HLM, dihydroxylated+hydrogenated-DLX (M10, 28.7%) is a primary metabolite, but not in MLM (2.86%). Meanwhile, demethylated-DLX (M4, 18.5%) is a major metabolite in MLM, but not in HLM (5.8%). The formation of multiple metabolites shows significant differences between HLM and MLM. The relative abundance of metabolite in HLM and MLM is presented in Fig. 4. Their structures were elucidated based on the exact mass and MS/MS fragments.

### Formation of DLX-NAc Adducts (M34–M37), 1-Naphthol-NAc-Sulfate (M38) and DLX-GSH Adduct (M39) in Mice.

**Fig. 5.**
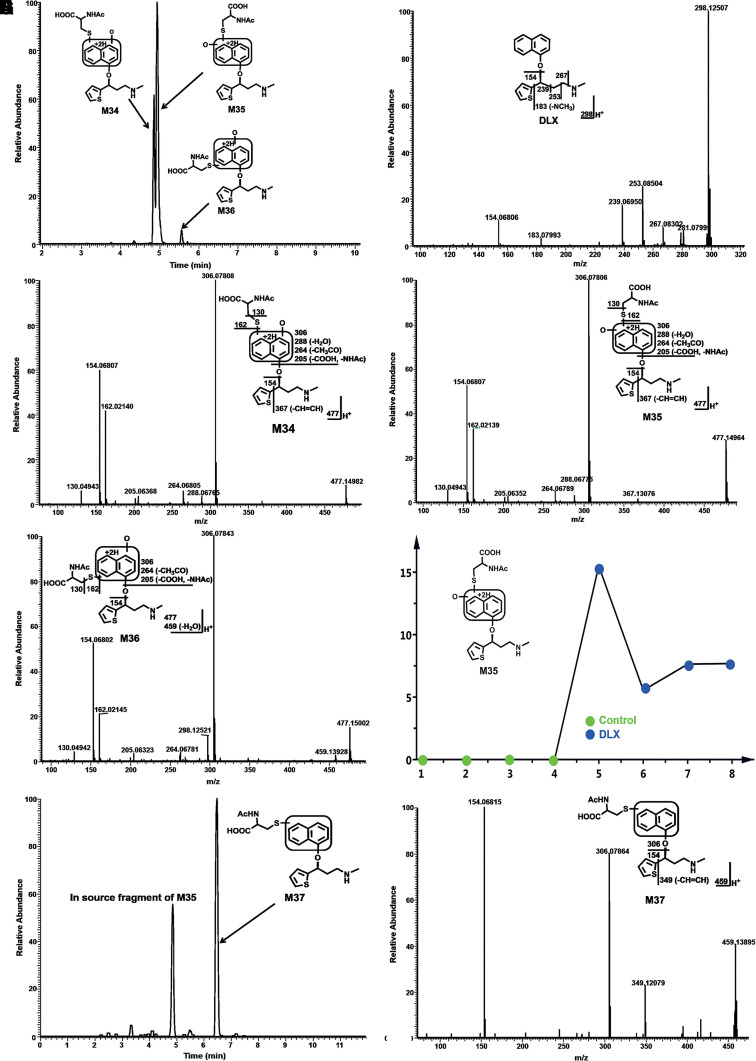
Formation of DLX-NAc adducts M34, M35, M36, and M37 in mouse urine and feces. Urine and feces from mice were collected continuously over the 18 hours after treatment (12 mg/kg, *p.o.*). The metabolites were analyzed using UHPLC-Q Exactive MS. Structural elucidations were performed based on accurate mass (mass errors less than 2 ppm) and MS/MS fragmentations. MS/MS was performed with collision energy ramping from 10, 20, 35 eV. The major fragmental ions are interpreted in the inlaid structural diagrams. (A) Chromatograms of metabolite M34–M36 in urine. (B) MS/MS of DLX. (C) MS/MS of M34. (D) MS/MS of M35. (E) MS/MS of M36. (F) Trent plot of M35. (G) Chromatograms of M37. (H) MS/MS of M37.

**Fig. 6.**
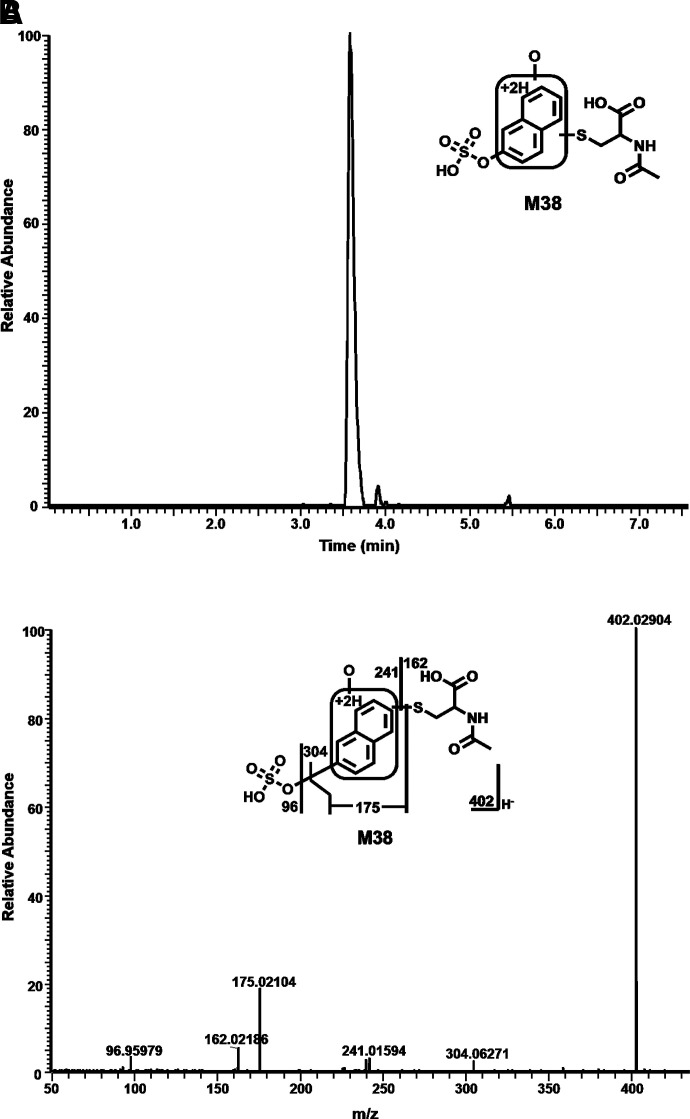
Formation of NAc-naphthol sulfate M38 in mouse urine. The urine samples were collected as described in Fig. 5. The samples were analyzed using UHPLC-Q Exactive MS in positive and negative modes. (A) Chromatograms of M38. (B) MS/MS of M38.

**Fig. 7.**
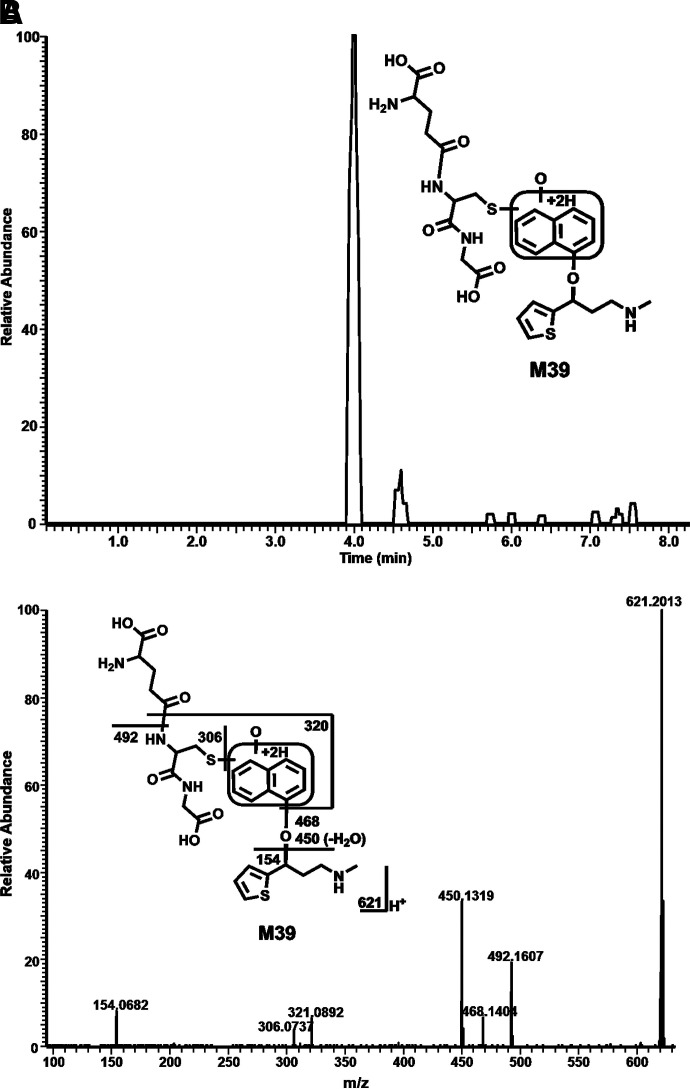
Formation of DLX-GSH adduct M39 in mouse liver. Liver samples were collected 90 minutes after the treatment of DLX (12 mg/kg, *p.o.*, *n* = 3). The samples were analyzed using UHPLC-Q Exactive MS in positive mode. (A) Chromatograms of M39. (B) MS/MS of M39.

**Fig. 8.**
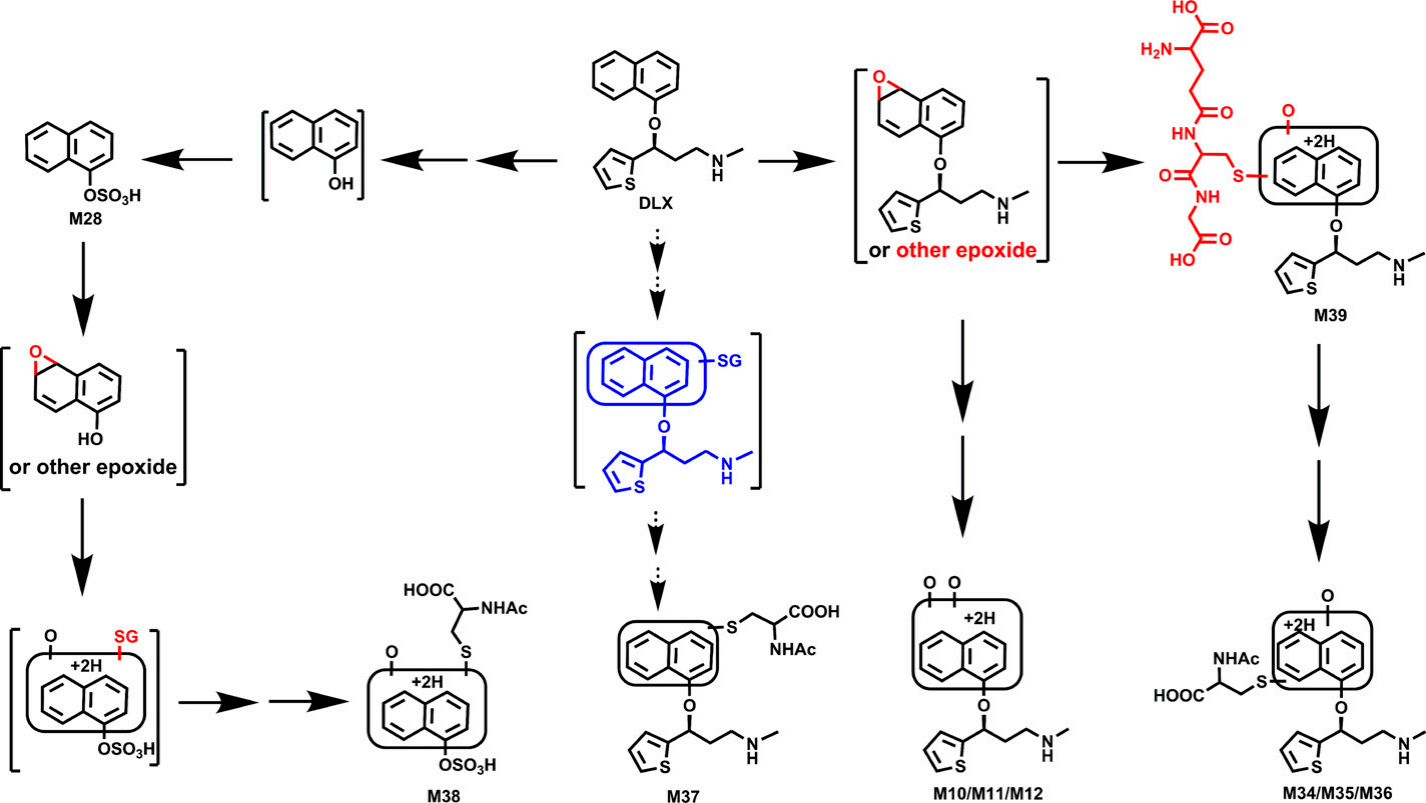
Proposed mechanism of the formation of dihydrodiol-DLX and DLX-NAc adducts. DLX was oxidized to epoxides, which reacted with GSH to form DLX-GSH adducts or hydrolyzed to dihydrodiol (M10–M12). M39 was further metabolized to DLX-NAc adducts (M34–M36) via multiple steps. The DLX-NAc adduct M37 could derive from one of DLX-NAc adducts (M34–M36) by loss of H_2_O, as the intermediate of direct DLX-GSH adduct in blue was not found in mouse liver. The formation of M38 requested multiple steps: (1) DLX was metabolized to generate 1-naphthol; (2) sulfation of 1-naphthol to form naphthol sulfate M28; and (3) epoxide of naphthol sulfate, followed by reacting with GSH and further metabolizing to NAc-naphthol+sulfate (M38). Alternatively, DLX-NAc adducts (M34–M36) were further degraded to NAc-naphthol adducts, which were sulfated to form the final metabolite M38.

Among these novel DLX metabolites, four DLX-NAc adducts (M34–M37) in urine and feces were observed in the positive mode. Metabolite M34 was eluted at 4.82 minutes (Fig. 5A), having a protonated molecule at *m/z* 477. The MS/MS of M34 produced the major fragment ions at *m/z* 306, 162, 154, and 130. The fragment ions were interpreted in the inlaid structural diagram (Fig. 5C). Metabolite M35 was eluted at 4.91 minutes (Fig. 5A), having a protonated molecule at *m/z* 477. The MS/MS of M35 produced similar major fragment ions as those of M34 at *m/z* 306, 162, 154, and 130. The fragmental ions were interpreted in the inlaid structural diagram (Fig. 5D). Metabolite M36 eluted at 5.53 minutes had a protonated molecule at *m/z* 477. MS/MS analysis of M36 produced fragment at *m/z* 306, 162, 154, and 130. The fragmental ions were interpreted in Fig. 5E. Figure 5F is a representative trend plot of M35, which suggested DLX-NAc adduct (M35) was only presented in the urine from DLX-treated mice. Metabolite M37 was eluted at 6.48 minutes (Fig. 5G) having a protonated molecule at *m/z* 459. The MS/MS of M37 produced the major fragment ions at *m/z* 349, 306, and 154. The fragmental ions were interpreted in Fig. 5H. Compared with MS/MS of DLX (Fig. 5B), fragment at *m/z* 154 in metabolites M34–M37 indicated that NAc was attached to the naphthol ring. M35 and M36 showed the similar MS/MS pattern with that of M34, suggesting that the NAc motif was just linked to a different position of naphthol ring. In the analysis of data from negative mode, 1-naphthol-NAc+sulfate (M38) was observed. M38 was eluted at 3.58 minutes (Fig. 6A), having a deprotonated molecule at *m/z* 402. The MS/MS of M38 produced the major fragment ions at *m/z* 304, 241, 175, 162 (-NAc), and 96 (-OSO_3_H). The fragmental ions were interpreted in Fig. 6B. In the liver from DLX-treated mice, DLX-GSH adduct (M39) was identified. M39 was eluted at 3.99 minutes (Fig. 7A) having a protonated molecule at *m/z* 621. MS/MS analysis of M39 produced fragments at *m/z* 492 (loss of glutamine), 468, 450, 321, 306, and 154. The fragmental ions were interpreted in the inlaid structural diagram (Fig. 7B). The fragment at *m/z* 154 in M39 indicated that GSH is linked to naphthol ring. The proposed mechanisms of DLX-NAc adduct formation are shown in Fig. 8.

### Role of CYP450s in the DLX Metabolism.

**Fig. 9.**
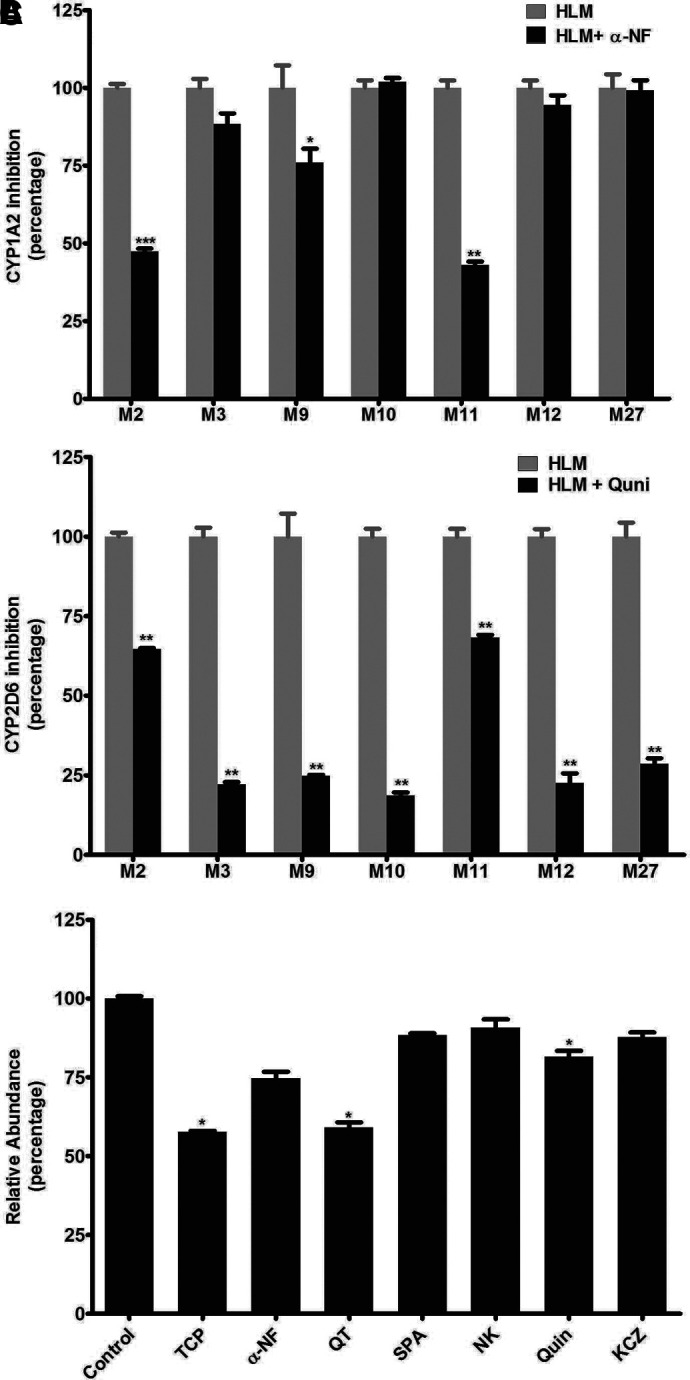
Role of CYP1A2 and CYP2D6 in DLX metabolism in human liver microsomes. *α*-NF (CYP1A2 inhibitor) and Quin (CYP2D6 inhibitor) were used in the inhibitory assay for the formation of DLX major metabolites in HLM. TCP (CYP2B6 inhibitor), *α*-NF, QT (CYP2C8 inhibitor), SPA (CYP2C9 inhibitor), NK (CYP2C19 inhibitor), Quin, and KCZ (CYP3A4 inhibitor) were used in the inhibitory assay for the formation of M4. The incubation conditions of DLX in HLM were detailed in experimental procedures. All samples were analyzed by UHPLC-Q Exactive MS. (A) Effects of *α*-NF on the formation of DLX major metabolites in HLM. The peak area of each metabolite from the incubation with HLM in the absence of *α*-NF was set as 100%. (B) Effects of Quin on the formation of DLX metabolite in HLM. (C) Effects of TCP, *α*-NF, QT, SPA, NK, Quin, and KCZ on the formation of M4 metabolite in HLM. The relative abundance of each metabolite from the incubation in HLM without inhibitors was set as 100%. *P < 0.05, **P < 0.01, ***P < 0.001. All the data are expressed as mean ± SEM (*n* = 3).

The role of CYP450s in the metabolism of DLX was determined by the inhibitory experiments in HLM with the corresponding potent chemical inhibitors and human cDNA-expressed P450s (control, CYP1A2, 2A6, 2B6, 2C8, 2C9, 2C19, 2D6, 2E1, and CYP3A4). Using recombinant human CYP450 enzymes, CYP2D6 was identified as the major enzyme responsible for the formation of DLX metabolites (M1–M3, M5, M6, M9, M12, and M27). Multiple enzymes contributed to the formation of M4. CYP1A2 was also involved in the production of M2–M4, M7, and M12 (Table 2). No single tested P450 was identified to be responsible for the formation of metabolites M8, M10, and M11. The role of CYP1A2 in the formation of DLX phase I major metabolites M2–M4, M9–M12, and M27 was verified by coincubation with *α*-NF (a potent CYP1A2 inhibitor) in HLM. The formation of M2, M3, M9, and M11 was suppressed by 53%, 17%, 24%, and 57% by *α*-NF at 6.0 *μ*M, correspondingly (Fig. 9A). *α*-NF has no effect on the generation of M10 and M12. The role of CYP2D6 in the formation of DLX metabolites was further demonstrated by coincubation of quinidine (a potent CYP2D6 inhibitor). The formation of M2, M3, M9–M12, and M27 was suppressed up to 36%, 78%, 75%, 81%, 32%, 74%, and 68% by Quin at 4.0 *μ*M, individually (Fig. 9B). The recombinant CYP450 enzymes studies indicated that multiple CYP450s are involved in the formation of demethylated-DLX (M4), including CYP1A2, CYP2B6, CYP2C8, CYP2C9, CYP2C19, CYP2D6, and CYP3A4. The role of these P450s in the formation of M4 metabolites was further determined by coincubation of the corresponding inhibitors (Fig. 9C). The formation of M4 was suppressed up to 42%, 25%, 42%, 14%, 12%, 22%, and 17% by TCP (CYP2B6 inhibitor at 10 *μ*M), *α*-NF (CYP1A2 inhibitor at 6.0 *μ*M), QT (CYP2C8 inhibitor at 30 *μ*M), SPA (CYP2C9 inhibitor at 4.0 *μ*M), NK (CYP2C19 inhibitor at 4.0 *μ*M), Quin (CYP2D6 inhibitor at 4.0 *μ*M), and KCZ (4.0 *μ*M, CYP3A4 inhibitor), correspondingly. This data indicated that all the tested P450s are involved in M4 formation, but CYP2B6 and CYP2C8 have relatively larger contribution in HLM.

**Fig. 10.**
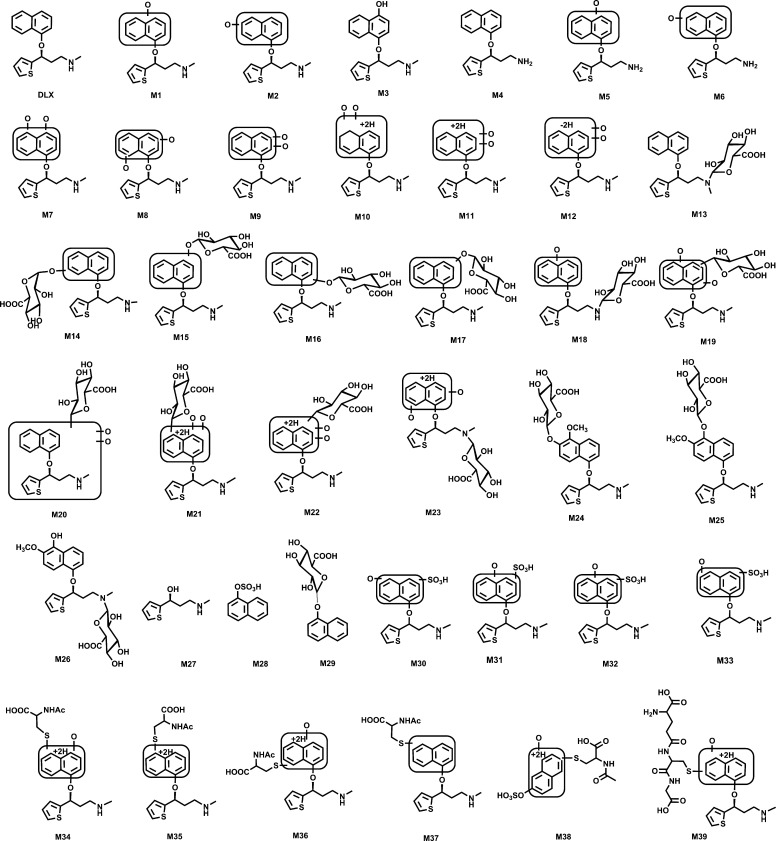
Summary of putative structures of DLX metabolites and adducts. All structures were determined based on the exact mass (mass error less than 2 ppm) and MS/MS fragments.

**TABLE 2:** Human recombinant CYP450 enzymes involved in the formation of DLX metabolites in vitro

	M1	M2	M3	M4	M5	M6	M7	M9	M12	M27
Control	0.0	0.0	0.0	0.0	0.0	0.0	0.0	0.0	0.0	0.0
CYP1A2	0.0	22.3	8.22	10.6	0.0	0.0	36.0	0.0	5.14	0.0
CYP2A6	0.0	0.0	0.0	0.0	0.0	0.0	0.0	0.0	0.0	0.0
CYP2B6	0.0	0.0	0.83	22.2	0.0	0.0	0.0	0.0	0.0	0.0
CYP2C8	0.0	0.0	0.0	38.5	0.0	0.0	0.0	0.0	0.0	0.0
CYP2C9	0.0	0.0	0.0	8.47	0.0	0.0	0.0	0.0	0.0	0.0
CYP2C19	0.0	0.0	0.0	84.5	0.0	0.0	0.0	0.0	0.0	0.0
CYP2D6	100.0	100.0	100.0	100.0	100.0	100.0	100.0	100.0	100.0	100.0
CYP2E1	0.0	0.0	0.0	0.0	0.0	0.0	0.0	0.0	0.0	0.0
CYP3A4	0.0	0.0	0.0	9.65	0.0	0.0	0.0	0.0	0.0	0.0

cDNA-expressed human CYP450s (control, CYP1A2, 2A6, 2B6, 2C8, 2C9, 2C19, 2D6, 2E1, and 3A4) were used to determine the role of individual CYP450 in DLX metabolism. All samples were analyzed by UHPLC-Q Exactive MS. The largest peak area of each metabolite from CYP enzymes was set as 100%. All data are expressed as mean (n = 2).

## Discussions

Systematical study of drug metabolism could offer the essential enlightenments concerning the efficacy and safety of a drug (Lin and Lu, 1997). Metabolomics has been successfully applied to the screening of stable and reactive metabolites in our previous studies (Li et al., 2011b; Li et al., 2012). In contrast to traditional LC-MS methods, metabolomic strategy could avoid the laborious process of predicting possible metabolites and readily identify the unexpected and unusual important metabolites (Li et al., 2011a; Li et al., 2011c; Li et al., 2011d). The metabolomics approach has the advantage over methods using radiolabeled drugs: (1) metabolomic approach is environment friendly as radiolabeled compounds are not needed. (2) metabolites lacking radiolabeled center can be discovered. The disadvantage is that the abundances of metabolites are relatively but not absolutely quantified, when standards of metabolites are not available. In this current study, we employed LC-MS-based metabolomic approaches to profile DLX metabolism in HLM, MLM, and mice. A total of 39 metabolites and adducts related to DLX were identified, including four DLX-NAc, one 1-naphthol- NAc+sulfate, and one DLX-GSH adducts, 13 phase I metabolites, 15 glucuronides, and five sulfates.

In humans, DLX was extensively metabolized to produce diverse oxidative and conjugated metabolites (Knadler et al., 2011). After a single oral dose of ^14^C-DLX, 72% of DLX and its metabolites were excreted in urine, whereas about 19% were excreted in feces based on radioactivity. Four circulating metabolites of DLX were observed: the glucuronide conjugate of 4-hydroxylated-DLX, sulfate conjugate of 5-hydroxy-6-methoxylated-DLX, 4,6-dihydroxylated-DLX, and 6-hydroxy-5-methoxylated-DLX. The most abundant metabolite in plasma is the glucuronide conjugate of 4-hydroxylated-DLX. Our current study showed that glucuronide conjugates of O+DLX (M14–M17) accounts for 93.8% of total metabolites (Fig. 2C) in mouse plasma, in which M14 (39.6%) and M15 (42.2%) are the most abundant metabolites. The data are consistent with human’s major metabolite, although their abundances are not qualitative. The second abundant sulfate conjugate in human plasma, 5-hydroxy-6-methoxylated-DLX, was not detected in mouse plasma. Novel metabolites, sulfate conjugate of 1-naphthol (M28), and glucuronide conjugate of 1-naphthol (M29) were also identified in the mouse plasma in negative mode (Fig. 3C). However, M28 was not reported in human plasma, probably because the molecule was ^14^C-labeled at the chiral center (Lantz et al., 2003). The 1-naphthol cleaved from DLX and its secondary metabolites lacked the ^14^C-label, which rendered them undetectable in human studies. In mouse urine, all the metabolites in plasma were observed, and glucuronide conjugates of O+DLX were the primary metabolites (81.9% of total metabolite in urine) as well (Fig. 2A). Sulfate conjugates of 5-hydroxy-6-methoxylated-DLX, the most abundant metabolites in human urine, was not found in the mouse urine either (Lantz et al., 2003). Trace amounts of sulfate conjugates of O+DLX (M30–M33) in mouse urine were detected (Fig. 3A). One sulfate conjugate of DLX in human urine was reported, but its abundance was undetermined as the peak was overlapped with glucuronide conjugate of 5-hydroxy-6-methoxylated-DLX. The dihydrodiol-DLX, as an unconjugated metabolite, was relatively abundant in human urine, whereas only tiny amounts of dihydrodiol-DLX (M10–M12) in mouse urine were detected (Fig. 2A). Instead, M3, a monohydroxylated-DLX, was the most abundant unconjugated metabolite in mouse urine (Fig. 2A). Our mouse studies revealed 17 metabolites in feces. The glucuronide conjugates of O+DLX (M13–M18), abundant in plasma and urine, were not present in mouse feces. In mouse feces, 4-hydroxylated-DLX (M3) was the most abundant metabolite, which was in line with human data. Most of the metabolites found in urine and plasma were also observed in mouse liver, among which M3 was the most abundant (Supplementary Fig. 1A). In the human study, one metabolite was uncharacterized in the urine, which could be identical to the newly identified glucuronide conjugate of DLX (M13) or glucuronide conjugate of desmethyl-DLX (M18) in mouse urine. To be noted, the abundance of metabolites in this current study are not quantitative. Our metabolic studies of DLX in HLM and MLM uncovered the large difference of dihydrodiol-DLX formation (M10–M11, Fig. 4). The species differences were also observed for the metabolites M1, M4, M12, and M27 between HLM and MLM (Fig. 4).

Generally, it is appreciated that the reactive metabolites play an important role in the development of idiosyncratic adverse drug reactions (Thompson et al., 2016). Commonly, NAc-conjugated adducts serve as one of the indicators for the formation of reactive metabolites in vivo. The formation of these metabolites imply that the reactive metabolites were produced in DLX metabolism, which could react with glutathione. NAc-adducts (M34–M37) were detected in mouse urine and feces, in which M34 and M35 were also observed in plasma. DLX-GSH adduct (M39) and DLX-NAc adduct (M35) were detected in mouse liver (Supplementary Fig. 1A). The NAc-naphthol+sulfate (M38) was present in mouse urine, plasma, and liver, but not in the feces. The mechanisms of the formation of M34–M37 were proposed as follows: briefly, DLX was oxidized to epoxides, then reacted with GSH to form DLX-GSH adducts (e.g., M39). The DLX-GSH adducts were further metabolized to DLX-NAc adducts (M34–M36) via a series of biotransformation steps (Jian et al., 2009). The DLX-NAc adduct M37 is likely derived from one of the adducts (M34–M36) by loss of H_2_O as the intermediate of direct DLX-GSH adduct (in blue) was not found in mouse liver (Fig. 8). Two dihydrodiols (M10 and M11) were observed in HLM/MLM and mice, which implied the epoxide formation during the DLX metabolism as generally dihydrodiols formed via the intermediate epoxide. Thus, M10–M12 could also serve as indicator of reactive metabolites formed from DLX metabolism. The proposed mechanisms are supported by previous studies, in which the presence of DLX-GSH conjugates (e.g., M39) were demonstrated by incubation of DLX in NADPH- and GSH-supplemented HLM (Wu et al., 2010; Chan et al., 2011).

The formation of NAc-naphthol+sulfate (M38) required multiple steps: (1) DLX was metabolized to generate 1-naphthol, accompanied by alcohol M27; (2) sulfation of 1-naphthol to form sulfate M28; and (3) epoxide of naphthol sulfate, followed by reacting with GSH and further metabolizing to NAc-naphthol+sulfate (M38). Both alcohol M27 and naphthol sulfate M28 were observed in urine, plasma, and liver (Figs. 2 and 3, Supplementary Fig. 1, and Table 1), supporting the occurrence of steps 1 and 2. Alternatively, DLX-NAc adducts (M34–M36) could be degraded to NAc-naphthol adducts, which were sulfated to furnish metabolite M38. Our data indicated that NAc conjugation exclusively occurred on the naphthalene ring, which is consistent with the in vitro studies (Wu et al., 2010; Chan et al., 2011). Although the formation of DLX-GSH adducts in HLM have been determined, DLX-NAc adducts identified in mice were not reported in human subjects. DLX-NAc adducts (M34–M37) retained the radio-labeled center, which should still have the radio activity. Theoretically, DLX-NAc adducts (M34–M37) could be detected if they were formed in human subjects. Thus, we proposed that the uncharacterized metabolite in human urine could be one of glucuronide conjugates (M13 or M18). It also might be one of the DLX-NAc adducts identified in mouse urine. As a couple of metabolites (M13, M18, M28, M29, M34–M37, and M38) have not been reported in human subjects using radio-labeled DLX, revisiting DLX metabolism in human subjects using LC-MS-based approaches will be valuable for comparing the clinical metabolic profile of DLX with that in mice.

The mechanisms of DLX-induced liver injury remain elusive. Previous limited studies speculated that the reactive metabolites from DLX might contribute to its adverse effects. Our studies indicated that CYP1A2 and CYP2D6 mainly contributed to the formation of DLX phase I metabolites (Table 2 and Fig. 9), which is in line with previous studies (Wu et al., 2010; Chan et al., 2011). To be noted, the formation of dihydrodiols (M10 and M11) via epoxide intermediates required multiple enzymes, since single P450 enzyme tested in our study did not produce metabolites M10 and M11. Our inhibitory experiments suggested that CYP2D6 was involved in the formation of M10 and both CYP1A2 and CYP2D6 in M11 generation. Wu et al. also reported that the formation DLX-GSH adducts were mainly catalyzed by CYP1A2 and CYP2D6 (Wu et al., 2010; Chan et al., 2011). Clinically, coadministration with fluvoxamine (CYP1A2 inhibitor) or paroxetine (CYP2D6 inhibitor) increase the peak plasma concentration, systemic exposure, and half-life of DLX (Skinner et al., 2003; Lobo et al., 2008). If DLX toxicity is attributed to its reactive metabolites, increasing DLX metabolism should exaggerate its toxicity. Further studies are warranted to address the exact role of DLX metabolism and reactive metabolites in its toxicity.

In summary, this study identified 39 metabolites and adducts associated with DLX (Fig. 10), including five NAc- adducts, and one GSH- adduct. Moreover, we demonstrated that the roles of CYP450s in DLX metabolite formation and significant species differences of the formation of certain metabolites between HLM and MLM. To conclude, this study provided a global view of DLX metabolism and bioactivation in liver microsomes and mice, which could facilitate the deep understanding the mechanism of adverse effects and possible drug-drug interactions concerning DLX. Further studies are granted to illustrate the role of DLX metabolism in its adverse effects in vitro (e.g., primary hepatocytes) and in vivo (e.g., Cyp2d-null mice).

## Abbreviations

DLX: duloxetine
GSH: reduced glutathione
HLM: human liver microsomes
KCZ: ketoconazole
MLM: mouse liver microsomes
NAc: N-acetyl cysteine
*α*-NF: alpha-naphthoflavone
NK: nootkatone
OPLS-DA: orthogonal projection to latent structures-discriminant analysis
P450: cytochrome P450
Q Exactive MS: Q Exactive Hybrid Quadrupole-Orbitrap Mass Spectrometer
QT: quercetin
Quin: quinidine
UHPLC: ultra*-*high-performance liquid chromatography
SPA: sulfaphenazole
TCP: ticlopidine
